# Attentional bias in eating disorders

**DOI:** 10.1002/eat.20375

**Published:** 2007-05

**Authors:** Roz Shafran, Michelle Lee, Zafra Cooper, Robert L Palmer, Christopher G Fairburn

**Affiliations:** 1 Department of Psychiatry, Oxford UniversityOxford, England; 2 Leicester General HospitalLeicester, England

**Keywords:** attentional bias, eating disorders, dot probe task

## Abstract

**Objective:**

To examine the relationship between eating disorders and attentional biases.

**Method:**

The first study comprised 23 female patients with clinical eating disorders, women with high levels ofanxiety (*n* = 19), and three female normal control groups comprising low (*n* = 31), moderate (*n* = 21), or high levels of shape concern (*n* = 23). The second study comprised 82 women with clinical eating disorders and 44 healthy controls. All participants completed measures of eating disorder psychopathology and completed a modified pictorial dot-probe task.

**Results:**

In the first study, biases were found for negative eating and neutral weight pictures, and for positive eating pictures in women with eating disorders; these biases were greater than those found in anxious and normal controls. The second study replicated these findings and biases were also found for negative and neutral shape stimuli.

**Conclusion:**

It is concluded that future research should establish whether such biases warrant specific therapeutic interventions. © 2007 by Wiley Periodicals, Inc. Int J Eat Disord 2007

## Introduction

Cognitive biases have an important place in both the theory and treatment of eating disorders.^1^ From a theoretical perspective, confirmatory cognitive biases, including selective attention to disliked body parts, have been proposed to reinforce concerns about body shape and contribute to dietary restriction and restraint.^2^,^3^ Other theoretical models such as the ‘‘escape from self-awareness’’ account of binge eating^4^ emphasize the processing of more general ‘‘threat’’ information, and propose that patients with eating disorders avert their attention away from personal threats.^5^–^7^ According to the transdiagnostic model of eating disorders recently proposed by Fairburn et al.,^3^ anorexia nervosa (AN), bulimia nervosa (BN), and atypical eating disorders have many maintaining mechanisms in common including, it is hypothesized, attentional biases.

Despite the importance attached to attentional biases in theoretical accounts of the maintenance of eating disorder psychopathology, research on this topic is limited. The majority of studies have used the modified Stroop task and found increased emotional Stroop interference for words and pictures related to eating and shape.^8^–^12^ Patients with eating disorders have also been shown to demonstrate selective attention towards more general threatening words, particularly if they relate to threats towards the self (e.g., ‘‘failure’’).^5^,^6^ The significant limitations of the modified Stroop task as a measure of selective attention, however, are well documented^13^–^15^ and it is for this reason that other information processing paradigms such as the dot-probe task have been developed.^16^,^17^ The assumption on which this task is based is that participants are faster to respond to probes that appear in the same spatial location as the stimulus to which they are paying attention.

This dot-probe paradigm has been used extensively in patients with anxiety disorders demonstrating that anxious individuals and patients with anxiety disorders show an initial attentional bias towards threat^18^ and, in some of the anxiety disorders such as social phobia, they subsequently appear to show a bias away from threat.^19^–^21^ In theone studythat used the dot-probe task in 33 patients with AN and BN, it was found that patients with eating disorders, but not healthy controls, directed their attention away from positive shape and weight words, and there was a trend for them to direct their attention towards negative shape and weight words.^22^ No such biases have been found in restrained eaters^23^ although various biases have been shown in those with high bulimic attitudes,^24^ those with high levels of hunger^25^ and those who have fasted or have high levels of eating concerns.^26^

Taken together, these studies suggest that patients with eating disorders and those with concerns about eating, shape, and weight may have particular biases in the processing of information. However, the research has several limitations. First, the studies have used words as opposed to images relevant to eating, shape and weight, and this limits their ecological validity. Pictorial versions of the dot-probe task have used in other disorders^27^ and appear to be a more sensitive index of attentional bias than the word dot probe task.^28^ Second, the only study on patients did not include stimuli related to eating^22^ and neither did it include the most common diagnostic group -those with a diagnosis of ‘‘Eating Disorder Not Otherwise Specified’’ (or EDNOS^29^)oran appropriate anxiety comparison group. It is possible that any attention towards or away from specific stimuli could be attributed to the general level of anxiety given that such biases are known to exist in people with anxiety thus a comparison group of people with anxiety is desirable.^16^,^30^ Third, the traditional dot-probe task cannot establish whether attentionisbeing drawntowards threat or whether there is difficulty in disengaging attention from the threatening information.^31^–^33^

The overall goal of the present research was to establish whether patients with eating disorders show attentional biases for eating, shape, and weight related stimuli and, if so, whether such biases are specific to patients with eating disorders. Two studies were conducted using a pictorial dot-probe task. The first investigated the presence of attentional biases in patients with eating disorders, women with high, medium, and low levels of shape concern, and anxious controls. The second study was primarily a replication of the first using a larger patient sample. It was hypothesized that:

1. Attentional biases for eating, shape, and weight stimuli will (a) be present and (b) be stronger in patients with eating disorders than controls
2. The strength of attentional biases will be associated with the eating disorder psychopathology.

## Study 1

### Method

#### Participants

There were five groups of participants, all of whom had normal or corrected-to-normal vision. The first group (‘‘eating disorder’’ or ED) comprised 23 female patients; three with AN, six with BN, and 14 with EDNOS. Participants with AN and BN met DSM-IV^34^ diagnostic criteria based on ratings on the Eating Disorder Examination (EDE^35^). Participants with EDNOS had disturbed eating behavior that resulted in a clinical degree of secondary impairment in psychosocial functioning yet did not meet diagnostic criteria for AN or BN (see Fair-burn and Bohn, 2005,^29^ p. 698). The patients with eating disorders were recruited from primary and secondary care physicians as part of a treatment trial.

The control group of female patients with high levels of anxiety (‘‘Anxious’’; n = 19) were recruited from local community mental health teams and the local community, and the inclusion criterion was a score of 18 or above on the Beck Anxiety Inventory (BAI^35^). There were three female normal control (NC) groups comprising those with low (n = 31), moderate (n = 21), or high levels of shape concern (n = 23). These groups were recruited from the local community either using advertisements or by inviting women to participate who had taken part in research previously. These women were categorized on the basis of their Shape Concern subscale score on the EDE.^a^ Those in the ‘‘high’’ shape concern group had a score at least one standard deviation above the mean EDE Shape Concern subscale score, or a score of 5 or 6 on the EDE item ‘‘Dissatisfaction with shape’’. Scores in this range result in significant interference in psychosocial functioning (on the basis of previous research^36^). Those in the ‘‘moderate’’ shape concern group scored within one standard deviation (above or below), the mean on the EDE Shape Concern subscale score, and those in the ‘‘low’’ shape concern group scored at least one standard deviation below the mean EDE Shape Concern subscale score. None of the participants in the anxious or NC groups had a current or past clinical eating disorder. All participants were weighed and their height was measured.

#### Materials

##### Stimuli

An initial pool of 92 photographs relating to eating (n = 40), shape (n = 35), and weight (*n* = 17) were selected and rated. The images were either photographed specifically for the task, or sourced from photographic libraries or non-copy-righted images on the internet. Seventy-nine control pictures of animals were also obtained. Rating of the images was carried out by 18 clinicians and researchers working within two eating disorder units at Oxford. The raters were asked to indicate which category each picture was best represented by (eating, body shape, body weight, animal, or none of these) and to indicate their emotional response to the image. On the basis of these ratings, a final pool of 18 ‘‘eating’’ pictures (6 positive, 6 negative, 6 neutral), 18 ‘‘body shape’’ pictures (6 positive, 6 negative, 6 neutral), and 6 ‘‘body weight’’ pictures (all neutral) were obtained and paired with a control picture matched for emotional valence. Examples of pictures within each eating-disorder stimuli type are included in Appendix 1. The ‘‘positive’’ eating images depicted ‘‘good’’ or low calorie food eaten in controlled circumstances (e.g. small amounts of celery with a fork). The ‘‘negative’’ eating images depicted high calorie foods, such as pizza or other similar food being eaten in an uncontrolled fashion (i.e., binge-like, with fingers). ‘‘Neutral’’ eating images contained items related to eating and the preparation of food, such as saucepans, cutlery, a menu, and the inside of a restaurant. ‘‘Positive’’ shape images included slim bodies or body parts such as thighs and stomachs. ‘‘Negative’’ shape images included plumper bodies or body parts such as thighs or stomachs. ‘‘Neutral’’ shape images included pictures of body parts less associated with weight and shape (e.g., eyes, noses, elbows). The weight images included pictures either of weighing scales, or people being weighed or weighing themselves. Animals were grouped according to how they were generally rated—for example, positive images included kittens and puppies, negative images included snakes and insects and neutral items included birds. Attempts were also made to match the picture pairs for complexity in terms of number of constituent components within each image pair.

All the photographs were color picture JPEG computer files. Each picture was edited to fit an upright rectangle measuring 13.5 cm × 9.5 cm. A personal computer was connected to a 51 cm monitor to display the pictures. The background color of the monitor was white.

##### Modified Dot-Probe Task

Participants were seated with their eyes 60 cm from the monitor and level with the center of the screen. Before each of the 84 trials, participants focused on a black number (0.8 cm in height) between 1 and 9 that was presented for 1,000 ms following the paradigm of Gotlib et al., 2004.^27^ They were instructed to say the number aloud to fixate them on the center of the screen to ensure that central fixation was achieved. The pairs of pictures (target (i.e., eating/shape/ weight) and non-target (animal) were presented on the left and right hand sides of the screen for a fixed period of 1,000 ms, following the paradigm of Mansell et al.^37^). The inside edges of the two pictures were separated by 14 cm horizontally. As the two images disappeared from the screen, a probe appeared immediately. The probe display consisted of a cross (X) 0.8 cm in height, in a location that corresponded to the center of one of the two pictures. Participants were required to press one of two buttons on a keyboard (marked with orange stickers), to indicate the position of the probe (B for left and N for right). The position of the eating-disorder relevant image and the position of the probe were balanced across trials so that each appeared in either location (to the right or left) with equal frequency, following the paradigm of Mogg and Bradley.^38^ After a response was made, the probe disappeared and the next presentation started immediately. Two trials were given as practice. Participants were tested individually and instructed to respond to the probe as quickly and as accurately as possible by an experimenter who was blind to group allocation. Each pair of images was presented twice. The order of pair presentation was randomized. After completing the dot probe task participants were shown each image again individually and asked to rate each in turn for emotionality (‘‘How it makes you feel when you look at it’’) on a scale from 3 (‘‘very negative’’) through 0 (‘‘completely neutral’’) to þ3 (‘‘very positive’’). The order in which images appeared for rating was randomized.

#### Measures

All participants completed all the measures.

##### Eating Disorder Examination (EDE^39^)

This is the ‘‘gold standard’’ interviewer-based measure of eating disorder psychopathology. It assesses the main behavioral features of eating disorders (e.g., dietary restriction, episodes of binge eating, vomiting) and generates subscales that assess dietary restraint, eating concern, shape concern, and weight concern. The global score is the mean of the four sub-scales. The EDE uses a 7-point forced-choice rating scheme for items comprising these subscales. The 28-day frequencies of key eating disorder behavior are measured in terms of the number of days on which each particular form of behavior occurs and the number of episodes. The EDE can be used reliably^40^ with test re-test coefficients in the region of .7 or higher, and inter-rater reliability coefficients of approximately .9. Convergent and divergent validity is also good.^41^

##### Beck Depression Inventory II (BDI-II^42^)

This is a 21-item self-report instrument for measuring the severity of depression and assesses symptoms corresponding to the diagnostic criteria for depressive disorder specified in DSM-IV.^34^ Each item is scored on a four-point scale ranging from 0 to 3, and the total score is obtained by summing the ratings for each item. Its reliability and validity are well established.^42^

##### BAI 35

This is a 21-item self-report instrument which assesses the severity of anxiety, and was especially designed to minimize its relationship with depression. Each item is rated on a four-point scale ranging from 0 to 3, and a total score is generated by summing the items. The scale is internally consistent, has good test-retest reliability^43^ and has been shown to be ecologically valid.^44^

#### Statistical Analysis

The data analysis was based on reaction times (RTs) for correct responses. The mean percentage of data lost owing to errors ranged from 1% (high shape concern control group) to 3% (eating disorder group). The difference in errors made across groups was not significant (*F*(4,112) = 2.26, *p* > .05). Following procedures from previous research,^45^ latencies of <200 ms and more than 2,000 ms were excluded and for each participant outliers were removed by excluding detection latencies that were beyond two standard deviations from their mean (i.e. from each individual’s mean RT across all stimuli). Following MacLeod et al.,^46^ a bias score was calculated for each stimulus type in the following way: bias score = RT when target and probe were in opposite positions minus RT when target and probe were in the same position. Positive values reflect that participants were quicker to respond to the probe when it appeared in the same location as the target (eatingdisorder relevant) image. Negative values reflect that participants were slower to respond to the probe when it appeared in the same location as the target (eating-disorder relevant) image.

Initially, biases within the eating disorder group were investigated via a series of initial 3 (valence; positive, negative, neutral stimuli) × 2 (probe position; same as target, opposite to target) repeated measures ANOVAs (followed up by paired t-tests). To compare bias scores across the groups, a series of 3 (valence; positive negative and neutral stimuli) × 5 (group; ED, anxious, low shape concern, moderate shape concern, and high shape concern) mixed ANCOVAs, (controlling for age, BMI and BDI-II scores) were carried out. The second hypothesis was investigated using correlational analyses and the third was investigated using series of one-way ANOVAs with planned contrasts (comparing the eating-disorder group with each control group in turn) to examine group differences in the way in which images were rated.

### Results

#### Demographic and Group Characteristics

The demographic and group characteristics are shown in Table 1. As expected, there were significant group differences on body mass index scores (BMI), the EDE, BDI-II and BAI. Tukey’s post-hoc analyses were conducted and the findings are shown in Table 1.

**TABLE 1:** Age, body mass index (BMI), eating disorder psychopathology, depression, and anxiety scores (and SDs) by group

	Eating Disorder	High Shape	Moderate Shape	Low Shape	Anxious	
	(*n* = 23)	Concern (*n* = 23)	Concern (*n* = 21)	Concern (*n* = 31)	(*n* = 19)	Group Differences
Age	22.17 (3.58)^a^	24.26 (5.63)^a,b^	27.90 (8.26)^b^	23.39 (6.69)^a,b^	26.26 (7.52)^a,b^	*F*(4, 112) = 2.77, *p* <. 05
BMI	21.79 (4.98)^a^	25.21 (3.29)^b^	25.04 (5.62)^a,b^	22.21 (2.34)^a,b^	22.33 (3.35)^a,b^	*F*(4, 112) = 4.00, *p* <. 005
Eating disorder examination Restraint	3.64 (1.51)d	2.36 (1.38)^c^	1.24 (1.14)^b^	0.63 (1.02)^a,b^	0.25 (0.74)^a^	*F*(4, 112) = 30.79, *p* <. 001
Shape concern	3.83 (1.31)^c^	3.36 (1.14)^b,c^	2.49 (1.01)^b^	1.07 (1.03)^a^	0.71 (0.68)^a^	*F*(4, 112) = 38.85, *p* <. 001
Weight concern	3.08 (1.49)^c^	3.07 (1.42)^c^	1.93 (1.24)^b^	0.90 (0.91)^a^	0.75 (0.84)^a^	*F*(4, 112) = 30.07, *p* <. 001
Eating concern	2.18 (1.29)^c^	1.60 (1.46)^c^	0.70 (0.79)^b^	0.27 (0.62)^a,b^	0.18 (0.49)^a^	*F*(4, 112) = 17.89, *p* <. 001
Beck depression inventory II	22.09 (13.84)^b^	6.21 (3.94)^a^	4.57 (4.09)^a^	7.45 (3.33)^a^	22.89 (11.62)^b^	*F*(4, 112) = 26.21, *p* <. 001
Beck anxiety inventory	16.78 (9.03)^b^	3.61 (2.84)^a^	3.86 (3.26)^a^	5.42 (3.39)^a^	25.11 (5.59)^c^	*F*(4, 112) = 70.43, *p* <. 001

Groups sharing the same superscript letter do not differ from each other (*p* < 0.05). Those with different superscript letters differ significantly (*p* < 0.05 or greater).

##### Hypothesis 1

Attentional biases for eating, shape and weight stimuli will (a) be present and (b) be stronger in patients with eating disorders than controls.

#### Eating Stimuli

1. Within the eating disorder group, there was no main effect of valence on RTs (*F*(2,21) = .92, *p* = .41). There was a significant effect of probe position on RTs (*F*(1,22) = 6.30, *p* = .02). Inspection of the means indicated that over all trials patients with eating disorders were slower to respond to the probe when the probe and target pictures were presented in the same location than an opposite location (RT = 513.81 and 488.20 ms respectively). There was also a significant valence x probe position interaction (*F*(2,21) = 10.92, *p* = .001). Paired t-tests indicated that for negative eating stimuli, patients with eating disorders were significantly quicker to respond to the probe when it was in the same location as the target picture than an opposite location (RTs = 473.96 and 531.52 ms respectively; *t*(22) = 2.29, *p* = .03) whereas the opposite pattern was found for positive eating stimuli i.e., they were significantly slower to respond to the probe when it was in the same location as the target picture than when it was in an opposite location (RTs = 557.45 and 426.66 ms respectively; *t*(22) = 5.26, *p* = .000). No bias was noted for neutral eating stimuli (*t*(22) = .02, *p* = .84).
2. Bias scores across the different groups are shown in Table 2. There were no significant main effects of valence (*F*(2,111) = 0.78, *p* = .46) or group (*F*(4,112) = 1.98, *p* = .16). There was a significant valence by group interaction (*F*(8,224) = 6.15, *p* = .000). This was followed up by univariate tests and planned contrasts (eating disorder group versus psychiatric controls; eating disorder group versus high shape-concern controls; eating disorder group versus low and mid shape-concern controls) for positive eating bias scores, negative eating bias scores and neutral eating bias scores. For positive eating stimuli, patients with eating disorders showed significantly more bias than the anxious controls (*p* < .001), high shape-concern controls (*p* < .001) and mid and low shape-concern controls (*p* < .001). For negative eating stimuli, patients with eating disorders showed significantly more bias than anxious controls (*p* < .005) and those with mid and low shape-concern (*p* < .001). No group differences were noted for neutral stimuli.

**TABLE 2:** Interference (bias) scores (and SDs) for eating disorder and control groups*

	Eating Disorder (*n* = 23)	Anxious (*n* = 19)	Low Shape Concern (*n* = 31)	Moderate Shape Concern (*n* = 21)	High Shape Concern (*n* = 23)
Eating stimuli					
Positive	130.79 (119.36)	13.86 (76.54)*	27.58 (57.80)*	1.2 (61.37)*	4.08 (54.34)*
Negative	57.56 (120.40)	27.28 (38.00)*	38.75 (75.71)*	14.34 (59.86)*	13.39 (63.63)
Neutral	3.58 (91.05)	8.63 (94.51)	6.42 (74.83)	13.27 (92.81)	10.05 (93.37)
Shape stimuli					
Positive	5.87 (48.56)	14.35 (121.37)	2.21 (51.59)	8.27 (134.46)	5.91 (64.04)
Negative	18.31 (60.77)	6.45 (179.96)	2.74 (60.01)	19.24 (136.28)	16.23 (76.71)
Neutral	6.25 (61.61)	0.33 (118.84)	35.92 (110.63)	1.89 (60.73)	13.17 (81.41)
Weight stimuli					
Neutral	86.24 (129.11)	7.60 (129.94)*	14.65 (83.88)*	3.58 (181.18)*	4.47 (72.72)*

* Indicates significant difference from interference (bias) scores in eating disorder patients (using planned contrasts).

#### Shape Stimuli

1. Within the eating disorder group, RTs were similar, regardless of whether the probe appeared in the same or opposite location as the target image. There was no main effect of valence or probe position on RTs (*F*(2,21) = .19, *p* = .83 and (*F*(1,22) = .48, *p* = .50 respectively) and no significant valence x probe position interaction (*F*(2,21) = 1.36, *p* = .28).
2. The bias scores for shape stimuli were broadly comparable across the five groups (see Table 2). The mixed ANCOVA indicated that there were no significant main effects of valence (*F*(2,111) = .01, *p* = .99) or group (*F*(4,112) = .39, *p* = .82) and there was no significant valence by group interaction (*F*(8,224) = .51, *p* = .85), suggesting that biases for positive, negative and neutral shape stimuli did not differ across groups.

#### Weight Stimuli

1. Participants with eating disorders were quicker to respond when the target and probe appeared in the same location (RT = 437.38 ms) as compared with trials when the target and probe appeared in opposite locations (RT = 523.62 ms). A paired t-test indicated that this difference was statistically significant (*t*(22) = 3.20, *p* = .004).
2. Bias scores across groups are presented in Table 2. A one-way ANCOVA controlling for age, BMI and BDI-II scores indicated that there were significant group differences in bias scores (*F*(4,112) = 2.83, *p* = .028). Planned contrasts indicated that for neutral weight stimuli, patients with eating disorders showed significantly more bias than anxious controls (*p* < .05), high-shape concern controls (*p* < .05) and those with mid and low shape-concern (*p* < .005).

#### Attentional Bias Across Different Eating Disorder Diagnoses

**FIGURE 1:**
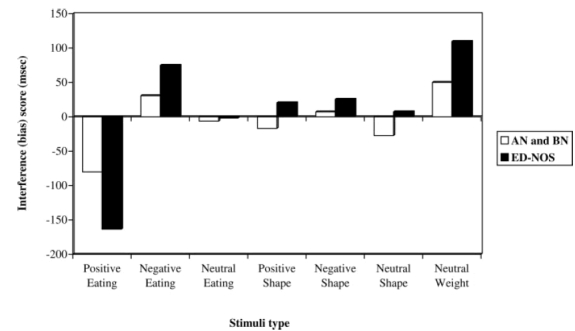
Interference (bias) scores for eating disorders patients by diagnosis.

As there were so few patients with ano-rexia-nervosa (*n* = 3), patients with AN and BN were compared with patients with EDNOS. Bias scores for each stimuli type for these two groups are presented in Figure 1. A series of independent-samples t-tests indicated that, consistent with theoretical accounts, attentional biases did not differ across diagnosis for any stimuli type (all dfs = 21, all *ts* < 1.9, (all ps > 0.07)).

##### Hypothesis 2

The strength of attentional biases will be associated with the eating disorder psychopathology.

Pearson correlation coefficients indicated a significant association between bias scores for negative eating stimuli and overevaluation of shape (*r* = .31, *p* = .001) and overevaluation of weight (*r* = .32, *p* = .001). Significant correlations were also found between bias for positive eating stimuli and overevaluation of shape (*r* = .22, *p* = .016), overevaluation of weight (*r* = .24, *p* = .008) and overevaluation of control over eating (*r* = .29, *p* = .002) indicating that the greater the core eating disorder psychopathology, the greater the bias with positive eating stimuli.

### Conclusion

The main finding of Study 1 is that patients with eating disorders were quicker to respond to a probe when it appeared in the same location as negative eating stimuli and neutral weight stimuli, and were slower to respond to a probe when it appeared in the same location as positive eating stimuli. These findings are largely consistent with the predictions made on the basis of theoretical accounts of attentional biases in eating disorders and previous research, with the exception of the lack of attentional bias for shape stimuli. It is of note, however, that similar findings were obtained in the only previous study using the dot-probe task with eating disorder patients^22^ and they indicated a need for replication using a larger sample. One possible explanation is for the finding is that the categorizations of ‘‘positive’’, ‘‘negative’’ and ‘‘neutral’’ were not made by patients. Additional unpublished data from our group indicate that there is a discrepancy between patient ratings of valence and those made by non-patients and that patients do not consider ‘‘positive’’ shape stimuli as positive; this may have contributed to the non-significant findings regarding the positive shape stimuli.

The lack of difference in biases found between those with a diagnosis of EDNOS and those with a diagnosis of AN or BN is consistent with the argument for a transdiagnostic approach to eating disorder psychopathology.^3^ However, the eating disorder group included very few patients with AN (n = 3) and hence the study was not able to determine whether attentional biases differ across the three main categories of eating disorders. Whether the attentional biases transcend diagnostic boundaries is an area for future research.

It was predicted that attentional biases would be stronger in patients with eating disorders than both anxious controls and women with high, moderate and low levels of shape concern. This hypothesis was supported and indicated that attentional biases to eating and weight stimuli cannot be explained by their high levels of shape concern or general psychopathology. The finding indicates that while normal and clinical samples of people with anxiety difficulties have common attentional biases,^30^ thesamecannotbesaidofpeoplewith high shape concern and clinical eating disorders. There was also support for the hypothesis that attentional biases would be correlated with eating disorder psychopathology, although the relationships were modest, not universal and sometimes counter-intuitive e.g., the finding that eating disorder patients tended to direct attention away from positive eating stimuli and yet there was positive correlation between attentional bias towards positive eating stimuli and eating disorder symptomatology. Given that the methods of reporting biases and the method of assessing the core psychopathology are so different, the inconsistencies are not altogether surprising and the findings stand in need of replication to establish their robustness.

One limitation of this study was that despite dealing with outliers in a standardized way, variance remained large within the sample particularly in the clinical group. However, Mogg and Bradley^38^ observe that RT variance and errors tend to be higher in clinical samples compared with normal samples. In addition, the sample size of patients was relatively small and the study may have been underpowered to detect some biases and associations. Since the findings from this study were of interest, the aim of Study 2 was to replicate the study with a larger sample of patients with clinical eating disorders.

## Study 2

### Method

#### Participants

The patient sample comprised 82 female patients who were referred by local clinicians for participation in a transdiagnostic eating disorder treatment trial. None had participated in Study 1. Patients were included if they met the following criteria: aged 18–65 years, judged to have a clinical eating disorder by one of three senior specialists in the field, body mass index between 16.0 and 39.9, able to attend for 20 sessions of outpatient treatment and judged to be safe to manage on an outpatient basis. Each patient completed the dot probe task immediately prior to starting treatment. The group comprised 50 patients with eating disorder not otherwise specified (ED-NOS; including six with binge eating disorder (BED)), 27 with BN and 5 with AN. These women were compared with 44 healthy control women who were recruited from the local community. Recruitment for controls was undertaken by advertising for female volunteers for participation in ‘‘body-image related’’ research throughout the Oxfordshire region. Participants were included if they met the following criteria: female, aged 18–45 (age matched to patients with eating disorders), with no current depression and no current or past history of an eating disorder.

#### Materials

The stimuli and modified dot-probe task were the same as those used in Study 1. All participants completed the Eating Disorder Examina-tion—Self-report version questionnaire (EDE-Q^47^) which assesses eating disorder features over the last 28 days and is based on the EDE.^39^ It assesses the main behavioral features of eating disorders (e.g., dietary restriction, episodes of binge eating, vomiting and other compensatory behavior) and generates subscales that assess dietary restraint, eating concern, shape concern, and weight concern. It uses a 7-point rating scheme for each of the items that comprise these subscales. Frequencies of key eating disorder behaviors are measured in terms of the number of days on which each particular form of behavior occurs. The questionnaire has good reliability and validity.^48^ Patients with eating disorders also completed the interview version of the EDE^39^(see above).

#### Data Analysis

The data analysis was the same as described in Study 1.

### Results

#### Demographic and Group Characteristics

Demographic and group characteristics are shown in Table 3. Patients had a marginally lower body mass index (*t*(122) = 1.67, *p* = .051), and higher Restraint, Eating Concern, Shape Concern, and Weight Concern subscale scores on the EDE-Q (all ps < .01) than controls. Patients also made significantly more errors in the dot probe task (*t*(124) = 2.17, *p* < .05), although only correct responses were included in the analyses.

**TABLE 3:** Age, body mass index, eating disorder psychopathology, and accuracy scores (and SDs) for eating disorder and control groups*

	Eating Disorder (*n* = 82)	Controls (*n* = 44)
Age	25.87 (6.92)^a^	26.41 (6.50)^a^
Body mass index	21.59 (4.12)^a^	23.09 (3.92)^a^
Eating disorders examination		
Restraint	3.57 (1.41)	–
Eating concern	2.67 (1.44)	–
Shape concern	3.85 (1.28)	–
Weight concern	3.63 (1.26)	–
Eating disorders examination-questionnaire		
Restraint	3.59 (1.48)^a^	0.66 (0.84)^b^
Eating concern	3.76 (1.22)^a^	1.23 (1.02)^b^
Shape concern	4.67 (1.16)^a^	1.34 (1.06)^b^
Weight concern	4.29 (1.32)^a^	1.66 (1.12)^b^
% correct responses on the dot probe task	98.24 (5.30)^a^	99.98 (0.15)^b^

* Groups sharing the same superscript letter do not differ from each other (*p* > 0.05).Those with different superscript letters differ significantly (*p* < 0.001 for all Eating disorder examination-questionnaire scales and *p* < 0.05 for % of correct responses on the dot probe task).

##### Hypothesis 1

Attentional biases for eating, shape and weight stimuli will (a) be present and (b) be stronger in patients with eating disorders than controls.

#### Eating Stimuli

1. For patients with eating disorders, there was no main effect of valence on RTs (*F*(2,81) = 1.02, *p* = .37). There was a significant effect of probe position on RTs (*F*(1,82) = 4.55, *p* = .036) with patients significantly quicker to respond to the probe when the probe and target pictures were presented in the same location than opposite locations (RTs = 565.63 ms and 583.10 ms respectively; *t*(81) = 5.68, *p* < .000). There was also a significant valence by probe position interaction (*F*(2,80) = 44.04, *p* = .000). For the negative eating stimuli, patients responded significantly more quickly to the probe when it was in the same location as the target picture than the opposite location (mean RTs = 509.50 ms and 620.20 ms respectively; *t*(81) = 5.68, *p* = .000) whereas for positive eating stimuli the pattern was reversed (mean RTs = 616.92 and 548.28 ms respectively; *t*(81) = 8.13, *p* = .000). No difference was found between RTs in patients for the probe when it appeared in the same as opposed to the opposite location as the target picture for neutral eating stimuli (mean RT = 570.48 and 580.82 ms respectively; *t*(81) = .69, *p* = .49).
2. Actual bias scores for positive, negative and neutral eating stimuli are presented in Table 4. A significant valence by group interaction (*F*(2,123) = 24.49, *p* = .000) followed by a series of independent samples t-tests indicated that patients with eating disorders had greater bias scores for positive eating stimuli and negative eating stimuli than the controls (*t*(124) = 3.78, *p* = .0002 and (*t*(124) = 6.29, *p* = .000) respectively). Bias for neutral stimuli was low in both groups, and there were no group differences on this index (*t*(124) = .65, *p* = .52).

**TABLE 4:** Mean bias scores in eating disorder patients and controls (and SDs)*

Stimuli Type	Patients (*n* = 82)	Controls (*n* = 44)
Eating Stimuli		
Positive	−68.65 (109.41)^a^	−3.34 (44.66)^b^
Negative	110.70 (123.4)^a^	−11.69 (50.95)^b^
Neutral	10.35 (135.21)^a^	−4.45 (95.22)^a^
Shape Stimuli		
Positive	0.74 (152.42)^a^	−12.00 (62.29)^a^
Negative	89.97 (182.22)^a^	4.91 (76.54)^b^
Neutral	34.31 (139.78)^a^	−0.21 (58.41)^a^
Weight Stimuli		
Neutral	100.15 (157.88)^a^	−21.19 (65.61)^b^

* Groups sharing the same superscript letter do not differ from each other (*p* > 0.05). Those with different superscript letters differ significantly (*p* < 0.001 for positive and negative eating and neutral weight, and *p* < 0.05 for negative shape).

#### Shape Stimuli

1. With patients with eating disorders, there was no main effect of valence on RTs (*F*(2,80) = 1.48, *p* = .23) although there was a main effect of probe position (*F*(1,81) = 10.53, *p* = .0017). Patients were significantly faster to respond to the probe when it appeared in the same location as the target than the opposite location (RTs = 577.73 ms and 619.10 ms respectively; t(81) = 3.85, *p* = .0002). There was also a significant valence by probe position interaction (*F*(2,80) = 9.67, *p* = .0002) with participants being significantly quicker to respond to the probe when negative and neutral shape stimuli were in the same location as the target picture than the opposite location (*t*(81) = 4.47, *p* = .000 and t(81) = 2.22, *p* = .029 respectively). However, this was not the case for positive shape stimuli; t(81) = .44, *p* = .66)).
2. Comparing bias scores across groups (see Table 4), analyses indicated a significant main effect of valence (*F*(2,123) = 6.64, *p* = .0018) and group (*F*(1,124) = 5.78, *p* = .0177) with patients showing greater bias scores than controls with a marginally significant valence by group interaction (*F*(2,123) = 3.08, *p* = .05). Independent samples t-tests indicated that patients with eating disorders showed significantly greater bias for negative shape stimuli than controls (*t*(124) = 2.96, *p* = .0037) but did not differ from controls in terms of bias for positive shape stimuli (*t*(124) = .60, *p* = .55) or neutral shape stimuli (*t*(124) = 1.56, *p* = .121) (See Table 4).

#### Weight Stimuli

1. In patients, RTs to the probe were faster when the target and probe appeared in the same location than opposite locations (*t*(81) = 5.74, *p* = .000).
2. Actual bias scores for weight stimuli were significantly higher in patients with eating disorders than controls (*t*(124) = 4.87, *p* = .000).

### Conclusion

Study 2 involved a replication of Study 1 with a larger patient sample. As predicted, patients with eating disorders were faster to react to negative eating and neutral weight stimuli and slower to react to positive eating stimuli. However, unlike Study 1 and more consistent with theoretical predictions, participants in this study were also significantly faster to respond to the probe when it was in the same location as the target picture for negative and neutral shape pictures (for example, images of large thighs, or images of elbows). No bias was found for positive shape stimuli (e.g. slim figures). It may be the case that the pictures of shape need to be personally relevant to detect such biases and the positive images used did not have meaning for the participants.

The biases found were greater in patients with eating disorders than healthy controls with the exception of the bias for positive and neutral shape stimuli. Since there was no bias noted for positive shape stimuli in patients with eating disorders, it is not surprising that the degree of bias for positive shape stimuli did not differ across groups. Again, this pattern of findings may be related to the discrepancy between patient and non-patient ratings of the valence of eating disorder relevant stimuli.

## Conclusion

These studies aimed to investigate the nature of attentional biases in patients with clinical eating disorders. The findings demonstrated that patients with eating disorders have robust and reliable attentional biases relating to eating and weight stimuli, and indicate the evidence for a bias for shape related stimuli is less strong. In particular, patients with eating disorders were faster to react to a probe when it appeared in the same location as negative eating and neutral weight stimuli than when it appeared in the opposite location to the target stimuli and slower to react to a probe when it appeared in the same location as positive eating stimuli than when it appeared in the opposite location to the target stimuli. In Study 2, but not Study 1, participants with eating disorders were significantly quicker to respond to the probe when it was in the same location as the target picture for negative and neutral shape pictures (for example, images of large thighs, or images of elbows) but no bias was found for positive shape stimuli (e.g. slim figures). It may be the case that such biases are less robust and that the pictures of shape need to be personally relevant to detect such biases. In both studies, the eating and weight-related biases were greater in patients with eating disorders than controls, and there were some associations between degree of psychopathology and extent of attentional biases. However such associations were, on the whole, relatively modest and the question as to the exact nature of the relationship between the severity of psychopathology and extent of such biases remains intriguing. It is possible that for some patients, the two variables are closely associated such that having high psychopathology increases attentional biases and vice versa in a reciprocal relationship. However, for other patients, high degrees of psychopathology may not be particularly closely related to attentional biases.

The series of studies had a number of strengths. Notably, no similar studies have been conducted with such a sample, using a pictorial dot-probe task, using such state-of-the-art measures and interventions and using such a wide range of comparison groups including those with EDNOS, anxiety and high shape concern. However, the studies also had a number of limitations including the failure to use a paradigm that can determine whether faster responses to probes at target locations may reflect difficulty in disengaging from threat rather than vigilance to a threat. Such designs have been used in clinical samples with anxiety,^48^ dysphoria,^49^ and non-clinical samples.^50^ A further limitation of this research was that the stimuli were categorized according to the ratings of valence made by people without eating disorders and, as noted above, unpublished data from our group indicate that there is a discrepancy between ratings made by people with eating disorders and non-patient con-trols.^b^ It is suggested that future research examines attentional biases using categorizations made by patients, as these are likely to be the most ecologically valid.

Having established the presence of attentional biases, the question remains as to whether they act as independent maintaining mechanisms for which specific methods (such as attentional training) to address them may be warranted or whether they are expressions of the eating psychopathology and remit with successful treatment. Experimental manipulations of attentional bias in undergraduates indicate that inducing attentional biases towards shape and weight related stimuli increases body dissatisfaction^51^ whereas further research from our group indicates that they normalize after successful treatment.^52^ Experimental and therapeutic manipulations would help elucidate further the mechanisms that are hypothesized to contribute to the maintenance of eating disorder psychopathology and thereby contribute to the development of more efficient, personally relevant, interventions for our patients.

The participants in study 2 were recruited for a Trust-funded treatment experiment (046386). We are grateful to Helen Doll and two anonymous reviewers for helpful comments on an earlier version of this manuscript. We are also grateful to the following people for their assistance with data collection: Caroline Riley and Clare Farrell (study 1); Marianne O’Connor, Caroline Adams, Elizabeth Payne, Jocasta Webb (study 2).

## Figures and Tables

**Appendix 1:**
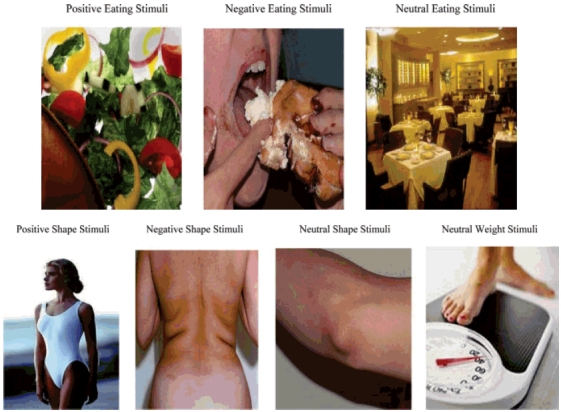
Examples of stimuli used in the dot probe task. [Color figure can be viewed in the online issue, which is available at http://www.interscience.wiley.com.]

## Footnotes

a Normative means; Shape Concern subscale = 2.45 (SD = 1.76). On the basis of a community sample of 243 women, mean age = 26.6 years, mean BMI = 23.7 (Unpublished data).

b Lee M, Shafran R. Methodological issues in the dot probe task.
